# Sensory Processing and Perceptual Alterations in Children Born Preterm: A Scoping Review

**DOI:** 10.3390/children13081096

**Published:** 2026-08-18

**Authors:** Clarissa de Oliveira Ruback, Camila Barros Moreira, Carla Trevisan Martins Ribeiro, Maria Dalva Barbosa Baker Meio

**Affiliations:** 1 Post-Graduation in Applied Clinical Research, Instituto Nacional de Saúde Da Mulher, Da Criança e do Adolescente Fernandes Figueira, FIOCRUZ (Fundação Oswaldo Cruz), Rio de Janeiro 21040-900, Brazil; clarissaruback@gmail.com; 2 Department of Physiotherapy, Instituto Nacional de Saúde Da Mulher, Da Criança e do Adolescente Fernandes Figueira, FIOCRUZ (Fundação Oswaldo Cruz), Rio de Janeiro 21040-900, Brazil; camilabarross.fisio@gmail.com (C.B.M.); carla.ribeiro@fiocruz.br (C.T.M.R.)

**Keywords:** prematurity, sensory development, child development, sensory integration, sensory disorders

## Abstract

**Highlights:**

**What are the main findings?**

- This scoping review identified frequent atypical sensory profiles in very preterm children born with gestational age of 32 weeks or less.
- The main affected areas were hyporesponsiveness, atypical tactile and vestibular reactivity, visual acuity, and visuospatial attention.

**What are the implications of the main findings?**

- The sensory abnormalities reported may have significant developmental repercussions and, without appropriate therapy, may result in more severe limitations or impairments in children born preterm.
- Including an occupational therapist in follow-up teams may improve the detection of abnormalities and support earlier interventions.

**Abstract:**

**Background and objectives:** Children born prematurely are more likely to have altered sensory profiles, which can affect family and school functioning. This scoping review aimed to analyze the relationship between prematurity and the development of atypical sensory profiles. **Methods:** A scoping review was conducted according to the Preferred Reporting Items for Systematic Reviews and Meta-Analyses extension for Scoping Reviews (PRISMA-ScR), including studies evaluating children aged 1 to 12 years who were born at a gestational age of ≤32 weeks. The data sources were PubMed, Scopus, and Web of Science. The search was limited to English-language studies published in the previous 10 years. The keywords “Sensory Processing”, “Child Development”, “Perception”, “Sensory Profile”, “Sensory Integration”, “Preterm”, “Infant Premature”, “Infant Extremely Low Birth Weight”, and “Infant Low Birth Weight” were used to search the databases. Articles with outcomes related to cerebral palsy, blindness, low vision, hearing impairment, behavioral disorders, and autism were excluded. After article selection, the atypical sensory profiles identified were organized into thematic categories and evaluated according to frequency of occurrence. **Results:** Seven articles were included and showed frequent atypical sensory profiles in very preterm children, especially in sensory processing, particularly hyporesponsiveness, and visual perception. **Conclusions:** These findings indicate altered sensory performance among very preterm children, which may negatively affect motor, cognitive, and behavioral development. Early sensory screening may improve detection and guide interventions to minimize adverse developmental outcomes.

## 1. Introduction

Prematurity is considered a significant public health challenge and one of the major causes of morbidity and mortality in the perinatal period. Premature birth interrupts fetal development, with repercussions for neuropsychomotor development, and is one of the most important risk factors for overall developmental impairments in these children [1], as well as an added risk factor for sensory processing difficulties.

Despite technological advances and neonatal care that have contributed to improved survival rates among preterm newborns in recent decades, morbidity remains high, especially among infants with lower gestational ages [2,3].

Lower gestational age is associated with an increased risk of motor, sensory, cognitive, and language impairments, highlighting the vulnerability of this population to long-term sequelae [4]. Preterm newborns, especially very and extremely preterm newborns, are at greater risk of sensory development abnormalities [5,6]. These abnormalities result from brain immaturity and early interruption of sensorimotor development, which occurs mainly during the last three months of pregnancy [4,7,8].

Prolonged stays in environments with many disorganized sensory stimuli, such as Neonatal Intensive Care Units (NICUs), with excessive noise, light, and touch, may overload infants’ sensory channels and lead to sensory dysregulation [9]. Furthermore, life-preserving procedures can limit contact with parents, hindering bonding and tactile sensory stimulation, which are essential for healthy emotional and behavioral development [9].

Sensory development is a neural process that enables the central nervous system to organize, process, and respond to stimuli from the environment and the body [8]. Appropriate sensory processing is essential for children to develop motor, cognitive, behavioral, and social interaction skills [9,10]. When sensory processing does not function properly, hypersensitivity, hyposensitivity, or heightened sensory seeking may arise, along with difficulties in behavior, eating, sleeping, and social interaction [9,10].

Children born very preterm are more likely to have altered sensory profiles, especially in the tactile, vestibular, and auditory channels, which can directly affect motor, emotional, and social development, with implications for family and school life [8,9]. Therefore, understanding the influence of prematurity on sensory development, particularly among children born very preterm, is crucial. A review of previous studies investigating these issues could provide a better understanding of sensory outcomes. The findings may reinforce the importance of occupational therapists working with follow-up teams to perform sensory screening in children born preterm. This study aimed to explore, through a scoping review, scientific research published in the previous ten years on sensory development outcomes in very preterm infants.

## 2. Materials and Methods

A scoping review was conducted because this design allows the broad gathering, synthesis, and analysis of findings from studies on the same topic, integrating theoretical and empirical literature. This type of review provides a comprehensive view of the phenomenon of interest, identifies knowledge gaps, and supports further studies [11,12]. A descriptive, qualitative scoping review was conducted from 25 November 2024 to 25 April 2025, following the Preferred Reporting Items for Systematic Reviews and Meta-Analyses extension for Scoping Reviews (PRISMA-ScR) checklist [13,14,15] and the five stages of the review process: definition of the research question; literature search; data collection, including definition of the information to be extracted from the studies; critical analysis of the included studies; and presentation of the review/synthesis of knowledge [14].

The PICo strategy was used: P, population (preterm newborns with a gestational age ≤ 32 weeks); I, interest (sensory development); and Co, context (prematurity). Initially, the study theme was established, focusing on sensory development in children born preterm. Accordingly, the population of interest was delimited, concentrating the review on children born at a gestational age of ≤32 weeks. The research question was then defined as follows: “How is the sensory development of preterm newborns with a gestational age of ≤32 weeks affected?”

Inclusion and exclusion criteria were defined for article selection. The inclusion criteria were studies published in English in the previous 10 years, since 2014, that evaluated sensory development in children born preterm with a gestational age of ≤32 weeks and aged 1 to 12 years, with at least one assessment at one year of age.

**Figure 1:**
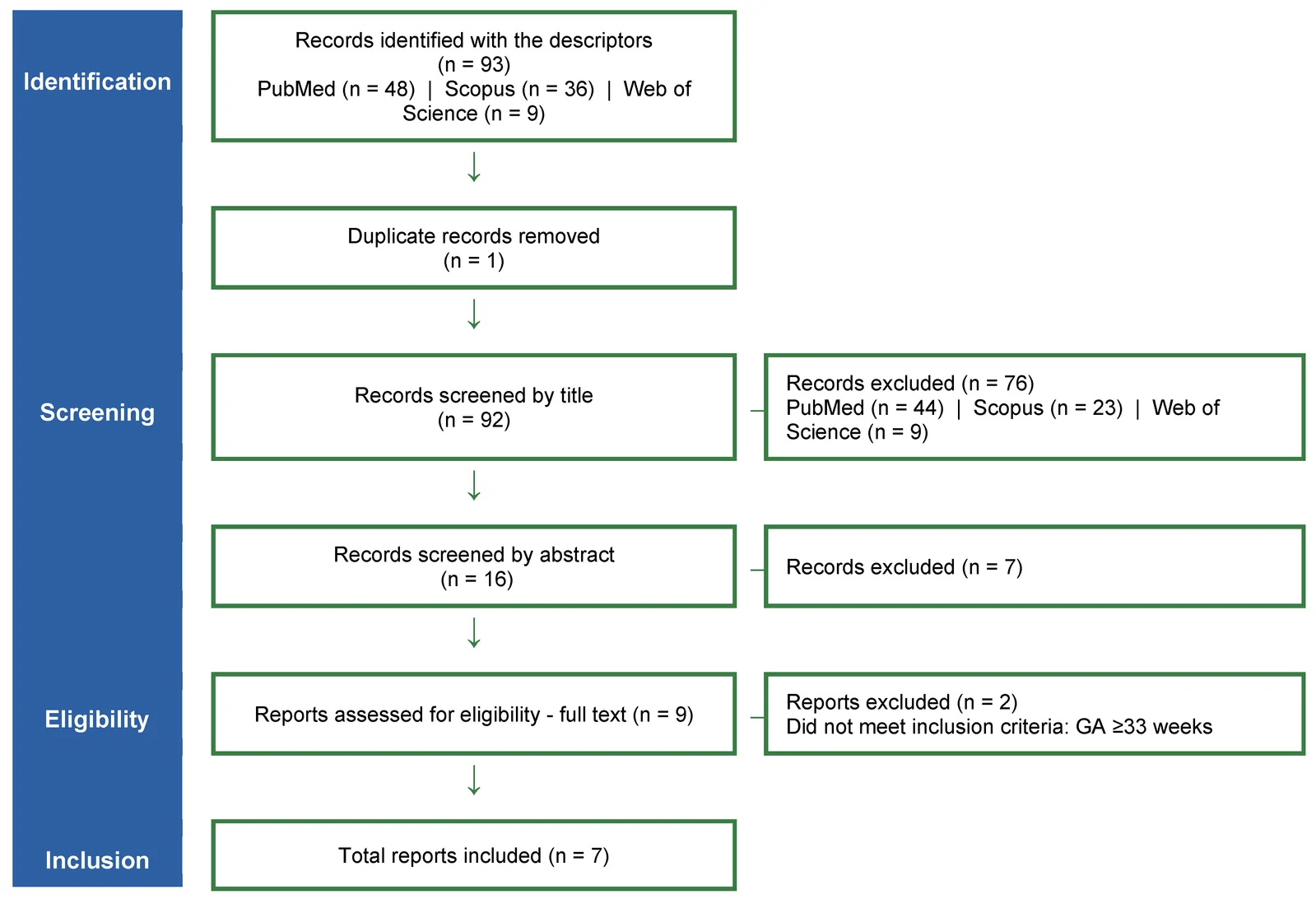
Description of the search based on the PRISMA flow diagram.

Case series, cross-sectional studies, cohort studies, case–control studies, and clinical trials were eligible, whereas articles unavailable in full text were excluded. To ensure comprehensiveness and relevance, the articles were required to establish explicit inclusion and exclusion criteria. Articles whose primary outcomes were cerebral palsy, blindness or low vision, hearing impairment, behavioral disorders, or autism were excluded, as were studies in which participants had any of these conditions. These conditions could act as confounding factors because they significantly impair child development, whereas this review focused on clinical sensory manifestations that might otherwise go unnoticed and on opportunities for early intervention. The third stage of this process involved identifying and locating sources that provided relevant information on the topic [14]. To this end, references from virtual libraries were selected by searching the following databases: PubMed, Scopus, and Web of Science. Figure 1 shows the selection process flowchart. To search for articles, descriptors that best represented the central objective of the research were selected from the Health Sciences Descriptors (DeCS), using “AND” to combine the descriptors in English (“Infant Extremely Low Birth Weight”; “Infant Low Birth Weight”; “Infant Premature”; “Child Development”; “Perception”; “Preterm infant”; “Sensory Integration”; “Sensory Processing”; “Sensory Profile”). The search strategy used for each database and the list of retrieved articles are shown in Supplementary Table S1.

The list of articles found in each database was evaluated by researchers CR and CB; initially, they jointly performed the selection based on titles and abstracts. The selected articles were then read in full, and the selection criteria were reapplied. After this second selection, researchers CR and CB read the articles and independently assessed them by completing the collection form developed for this review based on the references [12,14]. The researchers verified agreement in the assessments.

Disagreements between the independent reviewers were resolved by researchers MM and CT at the full-text stage to determine the final inclusion of each article; however, no agreement statistic was calculated. The analysis was conducted qualitatively, according to Hopia et al. (2016) [12] and Whittemore et al. (2005) [14], through careful reading of the included studies, extraction of relevant information, and organization into thematic categories.

After article selection, the reported sensory development abnormalities were extracted from the articles and entered into a spreadsheet together with the characteristics of the studies. This spreadsheet was developed for this review based on Hopia et al. (2016) [12] and Whittemore et al. (2005) [14] and included information on study type, characteristics of the included population, age at evaluation, instruments used for evaluation, and main study results. The reported sensory development abnormalities were evaluated according to frequency of affected domains in each study. Afterwards, they were grouped into thematic categories according to the main altered areas of sensory development: sensory processing alterations and visual perception alterations.

## 3. Results

After the literature review, seven studies were included in the final sample: four from Europe, two from North America and one from Canada. Five of the included articles were cohort studies, one was cross-sectional, and one was a case–control study. Sample sizes ranged from 32 to 160 participants. There was considerable variation between studies in the age at which outcomes were assessed (from 12 months to 9.2 years) and in the assessment instruments used (Table 1). However, the majority assessed children between the ages of 2 years and 5 years and 6 months: two studies at 2 years, two studies at 4 years and 6 months, and one at 5 years and 6 months. Evaluation of the frequency of the reported outcomes defined two groups of altered performance: sensory processing alterations and visual abnormalities.

### 3.1. Sensory Processing Alterations

Five articles reported alterations in sensory processing. Chorna et al. (2014) [5] investigated sensory reactivity in children born preterm (GA ≤ 30 weeks and BW ≤ 1500 g) at corrected ages (CA) of 4, 12, and 24 months. They found sensory alterations in some channels in 82% of children at 4 and 12 months of CA, mainly in deep tactile response and vestibular stimulation; these alterations were associated with worse neurodevelopmental assessments using the Bayley Scales of Infant Development, 3rd Edition (BSID-III), at 24 months of CA. Rahkonen et al. (2015) [16] evaluated extremely preterm infants (GA ≤ 28 weeks) at 2 years of CA regarding sensory processing and identified atypical sensory profiles in 52% of children, with low responsiveness as the most common pattern.

Crozier et al. (2016) [6] also evaluated sensory processing patterns at 4 and 5 years of age in very preterm children (GA ≤ 32 weeks), finding atypical sensory patterns in 46% of them; low responsiveness/sensation seeking was the most frequent profile. They reported that a lower Apgar score at 5 min and a longer stay in the Neonatal ICU were predictors of an atypical sensory pattern.

Ryckman et al. (2017) [17] analyzed children born preterm (GA ≤ 30 weeks) at term-equivalent age and at 4 and 6 years of age. Their evaluation found that half of the children had altered sensory processing and identified a significant association between atypical sensory profiles and early signs of stress and suboptimal reflexes observed in the neonatal period.

Bröring et al. (2018) [18] compared sensory processing between children born preterm (GA ≤ 32 weeks and/or BW ≤ 1500 g) and those born full term at 9.2 years of age. Children born preterm showed worse performance in somatosensory registration tasks and more difficulties in sensory modulation, such as hyporesponsiveness and hyperresponsiveness.

In summary, these studies showed frequent alterations in sensory processing in children born very preterm, emphasizing hyporesponsiveness, sensation seeking, and atypical reactivity, mainly in the tactile and vestibular domains. Chorna et al. (2014) [5], Rahkonen et al. (2015) [16], and Crozier et al. (2016) [6] reported these profiles in the children evaluated. Chorna et al. (2014) [5] reported that the worse responses were linked to lower GA. Crozier et al. (2016) [6] associated them with factors such as low registration of sensory stimuli and prolonged hospitalization in the Neonatal ICU. Other studies, such as those by Ryckman et al. (2017) [17] and Bröring et al. (2018) [18], indicated a relationship between neonatal stress, altered brain patterns, and persistent sensory impairments in childhood.

### 3.2. Visual Abnormalities

Two studies had more specific outcomes in the visual sensory channel. Geldof et al. (2014) [19] investigated visual sensory and perceptual functioning at 5.5 years of age in children born very preterm or with very low birth weight (GA ≤ 32 weeks and/or BW ≤ 1500 g) and identified poorer performance in visual acuity, inferior visual field, stereovision, and perceptual tasks in this group. Kooiker et al. (2019) [20] compared children born very preterm (GA between 26 and 32 weeks) regarding visual processing and visuospatial attention at 12 months of CA and found, in the preterm group, lower stimulus detection and longer latency to obtain a reaction, indicating dysfunction in attentional orientation and visual processing.

These studies reported alterations in visual perception in children born very preterm, including visual processing deficits, reduced acuity and visual field, and difficulties with visuospatial attention.

## 4. Discussion

This scoping review highlighted the vulnerability of children born prematurely, especially those with a gestational age of ≤32 weeks, to developing significant sensory processing alterations. Overall, altered sensory processing was observed in children born preterm, as identified through the assessments applied.

Assessment after 3 years of age is preferable because greater neurological maturity and behavioral stability favor more accurate and reliable observations [21,22,23]. Before this age, the sensory system is still developing, and typical behaviors show considerable variability, hindering the clinical identification of sensory dysfunctions [24]. Furthermore, the limited availability of standardized and validated instruments for children younger than 3 years compromises diagnostic accuracy [25]. From 3 years of age onwards, children show a more consistent functional repertoire, allowing better interpretation of sensory behaviors and greater clinical relevance of findings [24,26].

Atypical sensory profiles may be directly related to incomplete central nervous system development due to premature birth and early exposure to intensely stimulating environments, such as Neonatal ICUs, as suggested by Crozier et al. (2016) [6] and Ryckman et al. (2017) [17]. Vitale et al. (2021) [27] reinforce that these environments are marked by disorganized sensory stimuli and may adversely interfere with neurodevelopment, accentuating the risk of sensory dysregulation. Duerden et al. (2022) [7] demonstrated an association between painful neonatal procedures in preterm infants and thalamic growth—measured via MRI—as reflected in sensory behavior assessed at 4.5 years of age using the Short Sensory Profile (SSP). The results showed that a higher number of painful procedures were associated with slower thalamic growth in extremely preterm infants; furthermore, in both extreme preterm and very preterm infants, there was a positive association between thalamic volume and sensory assessment scores.

The prevalence of sensory alterations in preterm infants, as identified by Chorna et al. (2014) [5] and Ryckman et al. (2017) [17], suggests an association between prematurity and deficits in the tactile, vestibular, and auditory domains. Standardized instruments such as the Test of Sensory Function in Infants (TSFI) and the Infant/Toddler Sensory Profile (ITSP) were sensitive for the early detection of these impairments. The TSFI assesses sensory behavior from 4 to 18 months through child performance, whereas the ITSP uses parental reports to analyze sensory processing from birth to 36 months. According to Eeles et al. (2013) [28], both instruments have excellent predictive validity and, when applied together, allow more reliable identification of sensory disorders in early childhood.

Chorna et al. (2014) [5] also detected a significant association between altered sensory domains and motor outcomes at 2 years of age. The authors found that worse scores for adaptive motor function and oculomotor control at 4 and 12 months were predictive of adverse motor and language outcomes in the neurodevelopmental assessment at 24 months of CA. Geldof et al. (2014) [19] and Kooiker et al. (2019) [20] reported alterations in visual perception, including visual processing deficits, among very preterm children, which might hamper development. These findings are consistent with those of Eeles et al. (2013) [28], who examined infants born with GA < 30 weeks at 2 years of CA, assessing their sensory profile and neurodevelopmental outcomes, and found an association between sensory processing alterations and worse motor, cognitive, and language outcomes on the Bayley-III scale.

Recently, Yildiz et al. (2024) [29] also assessed sensory processing skills in preterm and full-term infants at 12 months using the TSFI and the Peabody Developmental Motor Scales-2 (PDMS-2). Their results showed that almost 30% of preterm children scored as “deficient” or “at risk” on the total TSFI score, with reactivity to deep tactile pressure, adaptive motor function, and visual-tactile integration showing the lowest scores in the preterm group. Preterm infants had lower total scores for locomotor and gross motor skills, with positive correlations between the gross and fine motor functions of the PDMS-2 and the total TSFI score, tactile response to deep pressure, and oculomotor control. This evidence shows that sensory processing difficulties in preterm infants may affect motor performance at 12 and 24 months of adjusted age.

The methodological heterogeneity of the included studies—with varying age ranges and different evaluation instruments—broadens the findings but limits evidence standardization. Although it was possible to identify the main altered sensory domains, the outcome definitions were heterogeneous.

Finally, social and environmental factors, such as parental education, have also been shown to be important predictors of sensory outcomes, as discussed by Chorna et al. (2014) [5]. These factors reinforce the need for integrated public policies that promote screening and early intervention, professional training, and family support, as advocated by Vitale et al. (2021) [27]. In this context, care for children born prematurely must be understood as a continuous and comprehensive process that extends beyond hospital discharge.

This review has some limitations. First, only English-language articles from developed countries were included, which may have excluded important results from other cultural settings, even though no search limits were applied in this respect. In addition, because we searched PubMed, Scopus, and Web of Science, our search may have missed studies published in journals not indexed in these databases or in gray literature sources. Nevertheless, the chosen databases encompass a large number of journals. Another limitation is the heterogeneity of the included studies, with different evaluation instruments and different ages at evaluation. The studies included in this review did not distinguish between gestational age groups, such as extreme preterm and very preterm infants; therefore, it was not possible to determine whether the sensory difficulties observed would be more pronounced in those born at lower gestational ages. Finally, the search terms were combined using the AND operator, which may have restricted the search. These limitations may restrict evidence standardization but do not diminish the importance of reporting atypical sensory profiles that might otherwise remain unnoticed.

## 5. Conclusions

This review showed that children born prematurely, especially at ≤32 weeks of gestation, are at higher risk of altered sensory processing, which may affect their motor, cognitive, and behavioral development. Although based on a small number of studies, the findings indicate that the impacts of prematurity may extend beyond physical outcomes and reach broader developmental dimensions. Therefore, an interdisciplinary and longitudinal therapeutic approach and the incorporation of early sensory screening into follow-up programs may be beneficial. The presence of an occupational therapist in the healthcare team could help in the detection of these atypical sensorial profiles. Interdisciplinary intervention programs and public policies that integrate continuous assessment and family support have potential clinical implications; therefore, they could minimize harm and promote comprehensive evaluation of the development of these children.

## Figures and Tables

**Table 1:** Description of the studies included in the review.

Authors/Year/Country	Study Type	Population	Control	Age at Evaluation	Instruments	Results	Limitations	Domain
Chorna et al., 2014—USA	Cohort	GA ≤ 30 weeks (median: 28 weeks (27–30)) and/or BW ≤ 1500 g (median: 1038 g (815–1308) Eligible: 79 Included: 72 Losses: 7	No	2 years CA	TSFI, BSID-III	82% showed sensory alterations. Worse responses linked to lower GA, white matter lesions, and caregiver education. Abnormal reactivity predicted worse neurodevelopment at 2 years of age.	Assessment performed in a clinical setting, which may not reflect habitual home behavior; follow-up loss at 2 years (infants > 30 weeks); reduced sample size limiting statistical power.	Multiple domains (TSFI): tactile deep pressure, adaptive motor function, visual-tactile integration, ocular-motor control, vestibular stimulation
Geldof et al., 2014—Netherlands	Cross-sectional	116 children born VLBW preterm (GA ≤ 32 weeks—30.1 weeksrange 28.1–31.6 weeks; and/or BW ≤ 1500 g)—1245 g, range 965–1480 g) 73 children born at term (GA > 37 weeks; BW > 2500 g) Selected at school. Losses: 60	Yes (n = 73), matched by sex, age at assessment, and parental schooling	5 years and 6 months	DTVP-2, WPPSI, vision and IQ tests	More visual sensory and perceptual deficits. Perceptual deficits were moderately linked to performance IQ and weakly linked to verbal IQ.	Refraction was assessed without cycloplegia, reducing precision; oculomotor measures may have lacked sensitivity for subtle dysfunctions; distinction between sensory and perceptual functions may be arbitrary.	Visual (sensory: acuity, visual field, contrast, color, stereovision; perceptual: visual coherence, spatial perception)
Rahkonen et al., 2015—Finland	Cohort	GA ≤ 28 weeks Eligible: 85 Included: 44 Losses: 41	No	2 years CA	GMDS, BSID-III, Hempel exam, MRI	52% had an atypical sensory profile. Sensory seeking was linked to brain injury; oral processing issues were linked to PDA surgery.	Small sample (n = 44) with few cases of atypical processing in each quadrant; no control group; sensory profile assessed only by parental report, without direct observation.	Multiple domains (ITSP): auditory, visual, tactile, vestibular, and oral—with focus on sensation seeking and oral processing
Crozier et al., 2016—Canada	Cohort; prospective and retrospective	GA ≤32 weeks Eligible: 160	No	4 years and 6 months	SSP	46% had atypical sensory processing. Underresponsiveness and sensation seeking were most common. Low Apgar scores and prolonged NICU stay were predictors.	Retrospective design; confounders such as procedural pain were not controlled; analyses were not adjusted for multiple comparisons; neonatal data were limited to first discharge; measurement was restricted to caregiver report.	Multiple domains (SSP): tactile, taste/smell, movement, auditory, visual–auditory—with emphasis on underresponsive/seeking patterns
Ryckman et al., 2017—USA	Cohort Prospective	32 children, GA ≤ 30 weeks	No	4 to 6 years	NNNS, SPA	50% had sensory processing disorder. It was linked to suboptimal reflexes and neonatal stress signs.	Reduced sample size (n = 32) with risk of Type I error; long interval between NICU discharge and assessment (4–6 years) without control of intermediate exposures; absence of normative data for the SPA.	Multiple domains (SPA): tactile, auditory, and visual—hyper/hyporesponsiveness and defensiveness
Bröring et al., 2018—Netherlands	Case–control	113 children: 57 children born preterm with GA ≤ 32 weeks (mean: 30.2 (1.8))**;** and/or BW ≤ 1500 g; (mean: 1293 (296)); 56 children born full term as a control group	Yes, matched by age and sex at assessment	9.2 years	MSIT, Sensory Profile, WISC-III	Children born preterm had somatosensory and modulation difficulties. Auditory–visual integration was preserved. Effects were small to medium (d = 0.30–0.56).	High attrition rate (50% of initial cohort); assessment in different environments (clinic vs. school); absence of white matter integrity data; use of parental questionnaire as a subjective measure.	Somatosensory/tactile (light touch registration, kinesthesia, graphesthesia) and multidomain sensory modulation; multisensory (audio-visual) integration
Kooiker et al., 2019—Netherlands	Cohort	123 preterm children (GA < 33 weeks)	No	12 months	Eye tracking	8–23% had visual processing and attention dysfunctions, linked to IRDS, IVH, and ventricular dilation.	Risk factors were obtained retrospectively, with possible incompleteness; small delayed-response groups (18–23 children); small control group (n = 38); possible underestimation of deficits in low-salience stimuli	Visual (visuospatial attention and processing—cartoon, contrast, form, motion, color stimuli)

TSFI, Test of Sensory Function in Infants; BSID-III, Bayley Scales of Infant and Toddler Development III; DTVP-2, Developmental Test of Visual Perception, 2nd ed.; WPPSI, Wechsler Preschool and Primary Scale of Intelligence; GMDS, Griffiths Mental Development Scale; SSP, Short Sensory Profile; NNNS, NICU Network Neurobehavioral Scale; SPA, Sensory Processing Assessment; MSIT, Multisensory Integration Test; WISC-III, Wechsler Intelligence Scale for Children, 3rd ed.; ITSP, Infant/Toddler Sensory Profile. MRI, Magnetic Resonance Imaging. GA, gestational age (in full weeks); IRDS, infant respiratory distress syndrome; BW, birth weight; CA, corrected age; PDA, patent ductus arteriosus; IVH, intraventricular hemorrhage.

## Data Availability

No new data were created or analyzed in this study. Data sharing is not applicable to this article.

## Supplementary Materials

The following supporting information can be downloaded at: https://www.mdpi.com/article/10.3390/children13081096/s1: PRISMA Checklist; Supplementary Table S1: List of the databases, search terms, and articles excluded after abstract and full-text reading.

## Abbreviations

The following abbreviations are used in this manuscript:

BSID-III	Bayley Scales of Infant Development, 3rd Edition
BW	Birth weight
CA	Corrected age
DeCS	Health Sciences Descriptors
EEG	Electroencephalogram
GA	Gestational age
g	Grams
ITSP	Infant/Toddler Sensory Profile
NICU	Neonatal Intensive Care Unit
OCEBM	Oxford Centre for Evidence-Based Medicine
PDMS-2	Peabody Developmental Motor Scales-2
TSFI	Test of Sensory Function in Infants

## References

[1] World Health Organization (2023). Premature Birth: Fact Sheet. World Health Organization. https://www.who.int/news-room/fact-sheets/detail/preterm-birth.

[2] Manuck T.A., Rice M.M., Bailit J.L., Grobman W.A., Reddy U.M., Wapner R.J., Thorp J.M., Caritis S.N., Prasad M., Tita A.T. (2016). Preterm neonatal morbidity and mortality by gestational age: A contemporary cohort. Am. J. Obstet. Gynecol..

[3] Stoll B.J., Hansen N.I., Bell E.F., Walsh M.C., Carlo W.A., Shankaran S., Laptook A.R., Sánchez P.J., Van Meurs K.P., Wyckoff M. (2015). Eunice Kennedy Shriver National Institute of Child Health and Human Development Neonatal Research Network Trends in Care Practices, Morbidity, and Mortality of Extremely Preterm Neonates, 1993–2012. JAMA.

[4] Hee Chung E., Chou J., Brown K.A. (2020). Neurodevelopmental outcomes of preterm infants: A recent literature review. Transl. Pediatr..

[5] Chorna O., Solomon J.E., Slaughter J.C., Stark A.R., Maitre N.L. (2014). Abnormal sensory reactivity in preterm infants during the first year correlates with adverse neurodevelopmental outcomes at 2 years of age. Arch. Dis. Child.-Fetal Neonatal Ed..

[6] Crozier S.C., Goodson J.Z., Mackay M.L., Synnes A.R., Grunau R.E., Miller S.P., Zwicker J.G. (2016). Sensory Processing Patterns in Children Born Very Preterm. Am. J. Occup. Ther..

[7] Duerden E.G., Mclean M.A., Chau C., Guo T., Mackay M., Chau V., Synnes A., Miller S.P., Grunau R.E. (2022). Neonatal pain, thalamic development and sensory processing behavior in children born very preterm. Early Hum. Dev..

[8] Machado A.C.C.P., Oliveira S.R., Magalhães L.C., Miranda D.M., Bouzada M.C.F. (2017). Sensory processing during childhood in preterm infants: A systematic review. Rev. Paul. Pediatr..

[9] Chen Y.C., Tsai W.H., Ho C.H., Wang H.W., Wang L.W., Wang L.Y., Wang H.H., Hwang Y.S. (2021). Atypical Sensory Processing and Its Correlation with Behavioral Problems in Late Preterm Children at Age Two. Int. J. Environ. Res. Public Health.

[10] Fritzsch B., Elliott K.L., Yamoah E.N. (2022). Neurosensory development of the four brainstem-projecting sensory systems and their integration in the telencephalon. Front. Neural Circuits.

[11] Khalil H., Peters M., Godfrey C.M., McInerney P., Soares C.B., Parker D. (2016). An Evidence-Based Approach to Scoping Reviews. Worldviews Evid.-Based Nurs..

[12] Hopia H., Latvala E., Liimatainen L. (2016). Reviewing the methodology of an integrative review. Scand. J. Caring Sci..

[13] Page M.J., McKenzie J.E., Bossuyt P.M., Boutron I., Hoffmann T.C., Mulrow C.D., Moher D. (2021). The PRISMA 2020 statement: An updated guideline for reporting systematic reviews. Int. J. Surg..

[14] Whittemore R., Knafl K. (2005). The integrative review: Updated methodology. J. Adv. Nurs..

[15] Tricco A.C., Lillie E., Zarin W., O’Brien K.K., Colquhoun H., Levac D., Moher D., Peters M.D., Horsley T., Weeks L. (2018). PRISMA Extension for Scoping Reviews (PRISMA-ScR): Checklist and Explanation. Ann. Intern. Med..

[16] Rahkonen P., Lano A., Pesonen A.K., Heinonen K., Räikkönen K., Vanhatalo S., Autti T., Valanne L., Andersson S., Metsäranta M. (2015). Atypical sensory processing is common in extremely low gestational age children. Acta Paediatr..

[17] Ryckman J., Hilton C., Rogers C., Pineda R. (2017). Sensory processing disorder in preterm infants during early childhood and relationships to early neurobehavior. Early Hum. Dev..

[18] Bröring T., Königs M., Oostrom K.J., Lafeber H.N., Brugman A., Oosterlaan J. (2018). Sensory processing difficulties in school-age children born very preterm: An exploratory study. Early Hum. Dev..

[19] Geldof C.J., Oosterlaan J., Vuijk P.J., de Vries M.J., Kok J.H., van Wassenaer-Leemhuis A.G. (2014). Visual sensory and perceptive functioning in 5-year-old very preterm/very-low-birthweight children. Dev. Med. Child Neurol..

[20] Kooiker M.J.G., Swarte R.M.C., Smit L.S., Reiss I.K.M. (2019). Perinatal risk factors for visuospatial attention and processing dysfunctions at 1 year of age in children born between 26 and 32 weeks. Early Hum. Dev..

[21] Dunn W., Little L., Dean E., Robertson S., Evans B. (2023). Sensory Profile 2: Manual.

[22] Eeles A.L., Spittle A.J., Anderson P.J., Brown N., Lee K.J., Boyd R.N., Doyle L.W. (2013). Assessments of sensory processing in infants: A systematic review. Dev. Med. Child Neurol..

[23] Lane A.E., Young R.L., Baker A.E., Angley M.T. (2010). Sensory processing subtypes in autism: Association with adaptive behavior. J. Autism Dev. Disord..

[24] Ben-Sasson A., Carter A.S., Briggs-Gowan M.J. (2009). Sensory over-responsivity in elementary school: Prevalence and social-emotional correlates. J. Abnorm. Child Psychol..

[25] Mulligan S., Case-Smith J., O’Brien J.C. (2014). Sensory integration: A collaborative approach to occupational performance and participation. Occupational Therapy for Children.

[26] Schoen S.A., Miller L.J., Mulligan S. (2021). Validity of the Occupational Performance Scale of the Sensory Processing Three Dimensions Measure. Am. J. Occup. Ther..

[27] Vitale F.M., Chirico G., Lentini C. (2021). Sensory Stimulation in the NICU Environment: Devices, Systems, and Procedures to Protect and Stimulate Premature Babies. Children.

[28] Eeles A.L., Anderson P.J., Brown N.C., Lee K.J., Boyd R.N., Spittle A.J., Doyle L.W. (2013). Sensory profiles obtained from parental reports correlate with independent assessments of development in very preterm children at 2 years of age. Early Hum. Dev..

[29] Yildiz R., Yildiz A., Zorlular R., Elbasan B. (2024). Relationship between sensory processing skills and motor skills in 12-month-old infants. Brain Behav..

