# Supplementary material for: Sensory Processing and Perceptual Alterations in Children Born Preterm: A Scoping Review
Source: Children (Basel). 2026 Aug 18;13(8):1096. doi: 10.3390/children13081096 (PMC13510308; doi:10.3390/children13081096)
Supplement: Supplementary file 1 [file children-13-01096-s001.zip › children-4383090-Supplementary Table S1.pdf]

Supplementary Table S1. List of the databases, search terms, and articles excluded after abstract and full-text reading.

DATABASE	SEARCH TERMS	APPLIED FILTERS	RESULTS
PubMed	"Infant Extremely Low Birth Weight" AND "Infant Low Birth Weight" AND "Infant Premature" AND "Child Development" AND "Perception" AND "Preterm infant" AND "Sensory Integration" AND "Sensory Processing" AND "Sensory Profile"	Last 10 years; English language; Abstract; Full text; Case Series, Cross-sectional, cohort, and case-control studies; infant (birth–32 months); preschool child (1–12 years). Search date: April 25, 2025.	**48 articles**
Scopus	"Infant Extremely Low Birth Weight" AND "Infant Low Birth Weight" AND "Infant Premature" AND "Child Development" AND "Perception" AND "Preterm infant" AND "Sensory Integration" AND "Sensory Processing" AND "Sensory Profile"	Last 10 years; English language; Abstract; Full text; Case Series, Cross-sectional, cohort, and case-control studies; infant (birth–32 months); preschool child (1–12 years). Search date: April 25, 2025.	36 articles
Web of Science	"Infant Extremely Low Birth Weight" AND "Infant Low Birth Weight" AND "Infant Premature" AND "Child Development" AND "Perception" AND "Preterm infant" AND "Sensory Integration" AND "Sensory Processing" AND "Sensory Profile"	Last 10 years; English language; Abstract; Full text; Case Series, Cross-sectional, cohort, and case-control studies; infant (birth–32 months); preschool child (1–12 years). Search date: April 25, 2025.	9 articles

AUTHORS/YEAR	TITLE	REFERENCE	REASON FOR EXCLUSION
Molloy et al., 2017	The contribution of visual processing to academic achievement in adolescents born extremely preterm	Molloy CS, Di Battista AM, Anderson VA, Burnett A, Lee KJ, Roberts G, Cheong JL, Anderson PJ, Doyle LW. The contribution of visual processing to academic achievement in adolescents born extremely preterm or extremely low birth weight. Child Neuropsychol. 2017 Apr;23(3):361-379. doi: 10.1080/09297049.2015.1118024.	Population: adolescents (outside the 1–12-year age range)
Kulmaganbetov et al., 2024	Associations between neonatal nutrition and visual outcomes in 7-year-old children born very preterm	Kulmaganbetov M, Leung M, Alsweiler JM, Black J, Bloomfield FH, Gamble GD, Harding JE, Jiang Y, Poppe T, Tottman AC, Wouldes TA, Thompson B; PIANO Study Group. Associations between neonatal nutrition and visual outcomes in 7-year-old children born very preterm. Ophthalmic Physiol Opt. 2024 Mar;44(2):347-355. doi: 10.1111/opo.13260.	Outcome: visual outcomes related to nutrition; sensory processing was not assessed
Dorta & Kychenthal, 2015	Spectral-domain optical coherence tomography of the macula in preterm infants	Dorta P, Kychenthal A. Spectral-domain optical coherence tomography of the macula in preterm infants treated with bevacizumab for retinopathy of prematurity. Ophthalmic Surg Lasers Imaging Retina. 2015 Mar;46(3):321-6. doi: 10.3928/23258160-20150323-04. PMID: 25856817.	Outcome: retinopathy of prematurity (major visual impairment – exclusion criterion)
Islam et al., 2018	Childhood language skills and adolescent self-esteem in preterm survivors	Islam UA, Poole KL, Schmidt LA, Ford J, Saigal S, Van Lieshout RJ. Childhood language skills and adolescent self-esteem in preterm survivors. J Child Health Care. 2018	Outcome: language and self-esteem; sensory processing was not assessed

		Mar;22(1):34-45. doi: 10.1177/1367493517739158. Epub 2017 Nov 6.	
van Veen et al., 2019	Visual perceptive skills account for very preterm children's mathematical difficulties	van Veen S, van Wassenaer-Leemhuis AG, van Kaam AH, Oosterlaan J, Aarnoudse-Moens CSH. Visual perceptive skills account for very preterm children's mathematical difficulties in preschool. Early Hum Dev. 2019 Feb;129:11-15. doi: 10.1016/j.earlhumdev.2018.12.018.	Outcome: mathematical performance; sensory processing was not the primary outcome
Herber-Jonat et al., 2014	Long-term outcome at age 7–10 years after extreme prematurity	Herber-Jonat S, Streiftau S, Knauss E, Voigt F, Flemmer AW, Hummler HD, Schulze A, Bode H. Long-term outcome at age 7-10 years after extreme prematurity - a prospective, two-center cohort study of children born before 25 completed weeks of gestation (1999-2003). J Matern Fetal Neonatal Med. 2014 Nov;27(16):1620-6. doi: 10.3109/14767058.2013.871699.	Broad neurodevelopmental outcome; the researcher did not specifically assess sensory processing
Rider et al., 2016	Test of visuospatial construction: validity evidence in extremely low birth weight children	Rider GN, Weiss BA, McDermott AT, Hopp CA, Baron IS. Test of visuospatial construction: Validity evidence in extremely low birth weight and late preterm children at early school age. Child Neuropsychol. 2016;22(5):587-99. doi: 10.1080/09297049.2015.1026889.	Outcome: isolated visuospatial construction; population included late preterm infants
Maitre et al., 2020*	Atypical multisensory processing in preterm infants	Maitre NL, Key AP, Slaughter JC, Yoder PJ, Neel ML, Richard C, Wallace MT, Murray MM. Neonatal Multisensory Processing in Preterm and Term Infants Predicts Sensory Reactivity and Internalizing Tendencies in	**Excluded after full-text reading:** population included preterm infants with GA >32 weeks, not meeting the

		Early Childhood. Brain Topogr. 2020 Sep;33(5):586-599. doi: 10.1007/s10548-020-00791-4.	inclusion criterion (GA ≤32 weeks)
Previtali et al., 2023*	Sensory Modulation Abilities in Healthy Preterm-Born Children: An Observational Study Using the Sensory Processing and Self-Regulation Checklist (SPSRC)	Previtali G, Lai CYY, Valvassori Bolgè M, Cavallini A, Nacinovich R, Piscitelli D, Purpura G. Sensory Modulation Abilities in Healthy Preterm-Born Children: An Observational Study Using the Sensory Processing and Self-Regulation Checklist (SPSRC). Biomedicines. 2023 Aug 21;11(8):2319. doi: 10.3390/biomedicines11082319.	**Excluded after full-text reading:** population included preterm infants with GA <37 weeks (not restricted to GA ≤32 weeks), not meeting the inclusion criterion (GA ≤32 weeks)

* studies excluded after full-text reading.
